# Macrophage polarisation under immune regulation as a therapeutic target for tendon-bone healing: multifactorial regulation and mechanistic insights

**DOI:** 10.3389/fimmu.2026.1737444

**Published:** 2026-02-03

**Authors:** Wanxue Wang, Liang Zhang, Yu Jiang, Dexi Cui, Kehao Hou, Shengquan Ren, Xia Zhao, Yingze Zhang, Ning Yu, Chao Qi, Kuishuai Xu

**Affiliations:** 1 Department of Sports Medicine, the Affiliated Hospital of Qingdao University, Qingdao, Shandong, China; 2 Department of Abdominal ultrasound, Affiliated Hospital of Qingdao University, Qingdao, Shandong, China; 3 Knee Preservation Centre, the Affiliated Hospital of Qingdao University, Qingdao, Shandong, China

**Keywords:** biomaterial, bone, macrophage, review, tendon

## Abstract

Recovery from a variety of surgical treatments, including arthroscopic rotator cuff repair and anterior cruciate ligament restoration, depends heavily on tendon-bone healing. There is mounting evidence that the polarisation of macrophages, namely M2 polarisation, is a crucial regulating factor in the repair of tendon-bone. Early tendon-bone repair is greatly aided by M1 macrophages, which have a pro-inflammatory nature. Long-term pro-inflammatory activity, however, seriously hinders the healing process. Therefore, one of the most important challenges in tendon-bone healing is to guide macrophages into the anti-inflammatory M2 phenotype. The effect of macrophage polarisation on tendon-bone healing is thoroughly investigated in this paper, along with methods for modifying macrophage polarisation. Importantly, it demonstrates how biomaterials control this process via a variety of signalling channels, providing fresh ideas for creating cutting-edge biomaterials (such as scaffolds, hydrogels, exosomes, etc.) that encourage tendon-bone mending by focusing on immune responses from macrophages.

## 1 Introduction

The tendon-bone interface, or TBI, is the connective tissue that runs between human tendons and bones. Its length ranges from 200 μm to 1 mm. The TBI is essential for preserving the healthy operation of the musculoskeletal system, despite its diminutive size. Four layers make up the TBI histologically: bone, calcified fibrocartilage, uncalcified fibrocartilage, and tendon. By facilitating the smooth transmission of stress from the tendon to the bone, this layered structure helps to avoid stress concentrations that could cause traumatic brain injury (1, 2). One of the main causes of re-tears may be the intricate structure of the TBI. In sports medicine, tendon-bone healing is still a hot topic, especially in TBI reconstruction. The TBI should ideally show signs of functional fibrocartilaginous repair. However, because of its structural intricacy, healing frequently takes the form of scar tissue, which makes it difficult to restore the normal structure. 20% of surgical failures have been attributed to high re-tear rates within two years following rotator cuff surgery, according to earlier studies (3). Another very common sports injury is an anterior cruciate ligament (ACL) rupture. However, following ACL reconstruction (ACLR), only a small percentage of patients regain full cruciate ligament function. With a cumulative failure rate of 11.9%, post-ACLR concerns include re-rupture and early knee osteoarthritis (OA). Inadequate tendon-bone union is still a major contributing cause, even beyond technical errors (4). The graft healing response, particularly the tendon-bone implant’s (TBI) osseointegration, determines the prognosis of ACLR (5).

Complete TBI regeneration and functional recovery are still difficult to achieve with current treatments, including physical therapy, medication, and standard surgical restoration. Current methods frequently concentrate on a single repair mechanism while ignoring complex elements like tissue remodelling and immunological responses, which results in slow healing processes that are prone to re-tears and delayed union.

Thus, it is now essential to properly take into account and control the immunological microenvironment to promote tissue regeneration during treatment in order to enhance therapeutic results. Macrophages are essential for tendon-bone repair, according to research. Macrophages can be categorised into several phenotypes according to their functions and triggering stimuli. M1 macrophages are predominant in the early phases of tendon-bone repair, whereas M2 macrophages take over in the later phases. In tendon-bone repair, these two unique macrophage phenotypes have essentially separate but equally significant functions. Studies show that M2 macrophages promote cartilage regeneration while M1 macrophages mainly aid in chondrocyte death and degenerative processes (6). Macrophages are important initiators and regulators of tendon-bone repair, according to mounting data. Modifying macrophage phenotypes, namely switching from pro-inflammatory M1 to anti-inflammatory M2 kinds, can improve tendon-bone fusion (1, 7). Furthermore, tendon-bone repair depends heavily on bone regeneration (8). Regenerated bone promotes the recovery of biomechanical qualities by facilitating the restoration of the four-layer structure at the tendon-bone interface, accelerating bone tunnel healing, and guaranteeing more robust tendon integration within the footprint region (9, 10).

Even while the role of macrophage polarisation in tendon-bone repair is becoming increasingly acknowledged, therapeutic research focusing on this mechanism is still in its early stages. In particular, there are no thorough evaluations and guiding systems for immunomodulatory therapeutic techniques based on biomaterials. Many biomaterials currently in use, including hydrogels, functionalized scaffolds, and nanomaterials, have shown great promise in controlling immune responses and facilitating tissue healing. Nevertheless, comprehensive reviews of these elements’ functions in macrophage polarisation are lacking in the literature. Therefore, this review thoroughly examines how biomaterials might be used to control macrophage polarisation and promote tendon-bone repair, providing fresh insights and treatment approaches for further study.

## 2 Macrophage phenotypes and polarisation

**Figure 1:**
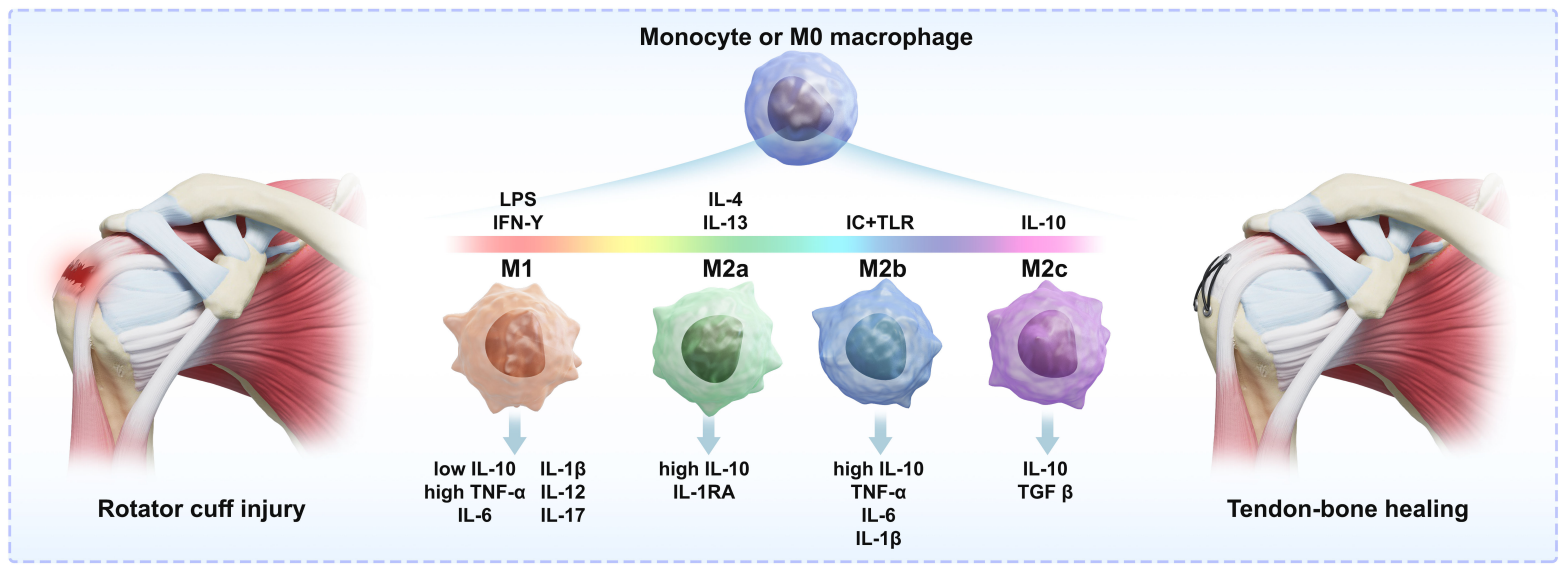
Macrophage polarisation and differentiation: Macrophages can be divided into two subtypes, M1 and M2, corresponding to different stages of regulatory processes. The transition of macrophages from M0 to M1 or M2 requires specific inducing factors, and the products of M1 and M2 in turn affect the cellular microenvironment, thereby influencing the process of tendon-bone healing.

All over the body, macrophages are responsible for protecting against pathogen invasion and preserving immunological homeostasis. As shown in **Figure 1**, in response to environmental stimuli, macrophages in various tissues polarise, resulting in the emergence of unique subtypes like M1 and M2 macrophages. Proinflammatory substances such as IFN-γ, LPS, and TNF-α stimulate M1 polarisation, whereas interleukin-4 (IL-4) produces M2 polarisation. Cytokines are essential for preserving bone homeostasis. There are several subtypes of M2-type macrophages: the M2a subtype, which is triggered by IL-4/IL-13, aids in tissue repair, whereas the M2c subtype, which is triggered by IL-10/TGF-β, reduces inflammation.M1-type macrophages release cytokines such as tumour necrosis factor (TNF), IL-6, and IL-12 and cause pro-inflammatory reactions. M2 macrophages, on the other hand, have anti-inflammatory properties and the ability to heal injured tissues. To help the host fight off infections, Macrophages first polarise toward the pro-inflammatory M1 phenotype in infected tissues. Anti-inflammation and tissue healing are facilitated by subsequent polarisation toward the M2 phenotype (11, 12).

Polarisation is governed by three main pathways: (1) Changing the epigenetic elements that affect the growth and survival of macrophages, (2) Changing the routes for cellular survival, and (3) Changing the tissue microenvironment or external variables (13). Depending on cytokine competition, exposure duration, and concentration, macrophage polarisation is dynamic and can happen at any stage of the inflammatory process (13). The application of macrophages in regulating the immune microenvironment has become increasingly widespread, extending beyond traditional cancer fields such as breast and prostate cancer, as well as diabetes, to gradually emerge as a significant factor in the repair of soft tissues, including tendon and bone healing (14–20).

## 3 Role of macrophages in tendon-bone healing

Tendon-bone healing comprises inflammatory, reparative, and remodelling phases. Macrophages exhibit distinct polarisation states during each stage: M1-type macrophages eliminate pathogens and necrotic tissue by releasing pro-inflammatory cytokines, reactive oxygen species, and matrix metalloproteinases, though their excessive activation may cause tissue destruction; whereas M2-type macrophages secrete anti-inflammatory factors, angiogenic factors, and ECM components to promote regeneration and scar formation (6, 21, 22). The first week post-injury constitutes the M1-dominant inflammatory phase, during which M1 cells primarily clear necrotic cells, degrade the ECM, and initiate the inflammatory response. However, sustained M1 polarisation inhibits osteoblast differentiation, delaying healing, necessitating the timely induction of M2 conversion. The 2–3 week period represents a critical proliferative phase for the M1-to-M2 transition. M2 macrophages secrete TGF-β and PDGF to promote MSC differentiation into fibroblasts or osteoblasts; they stimulate angiogenesis via VEGF and facilitate ECM deposition through collagen I and III, forming the fibrocartilaginous transition zone that determines TBI mechanical strength (23–26). Insufficient M2 cells during this phase leads to scar tissue formation and diminished mechanical properties. The subsequent 3 weeks to several months constitute the remodelling phase, where M2-type cells suppress MMPs via TIMP secretion, promote collagen I replacement of collagen III, and drive tissue maturation.

Macrophages persist throughout the healing process, secreting diverse inflammatory and growth factors to regulate cell proliferation, differentiation, and ECM formation. Research indicates that distinct macrophage subtypes promote MSC-mediated tendon-bone healing at each stage: M0 maintains homeostasis, while M1 and M2 sequentially participate in early inflammation and late-stage repair. The timing and duration of M1-mediated inflammation critically determine healing outcomes. Transient acute inflammation facilitates healing, whereas persistent chronic inflammation induces bone resorption by enhancing osteoclast activity and inhibiting osteogenesis. As the sole bone-resorbing cells, osteoclast differentiation is regulated by macrophage colony-stimulating factor and NF-κB ligand receptor activators (27). Thus, controlling the duration of M1 inflammation is paramount. *In vivo* macrophage depletion studies further confirm that early-stage macrophage deficiency most adversely impacts tendon-bone union. MSCs possess immunomodulatory functions, enabling their recruitment to injury sites by inflammatory macrophages. During repair, they mediate M1-to-M2 phenotype conversion to promote healing (28). This review thus explores three regulatory strategies: (1) promoting M1 polarisation in the early inflammatory phase while suppressing M1 in the late phase; (2) promoting M2 alone in the late inflammatory phase; (3) simultaneously suppressing M1 and promoting M2 to optimise tendon-to-bone healing.

## 4 Factors and mechanisms promoting M1 macrophage polarisation to enhance tendon-bone healing in the early inflammatory phase

### 4.1 Nanomaterials—copper-doped mesoporous silica nanospheres

Research indicates that M1 macrophages predominate during the initial phase of tendon-bone junction injuries. The pro-inflammatory cytokines they secrete and the reactive oxygen species they generate exacerbate the inflammatory response, thereby establishing a pro-inflammatory immune microenvironment.

**Figure 2:**
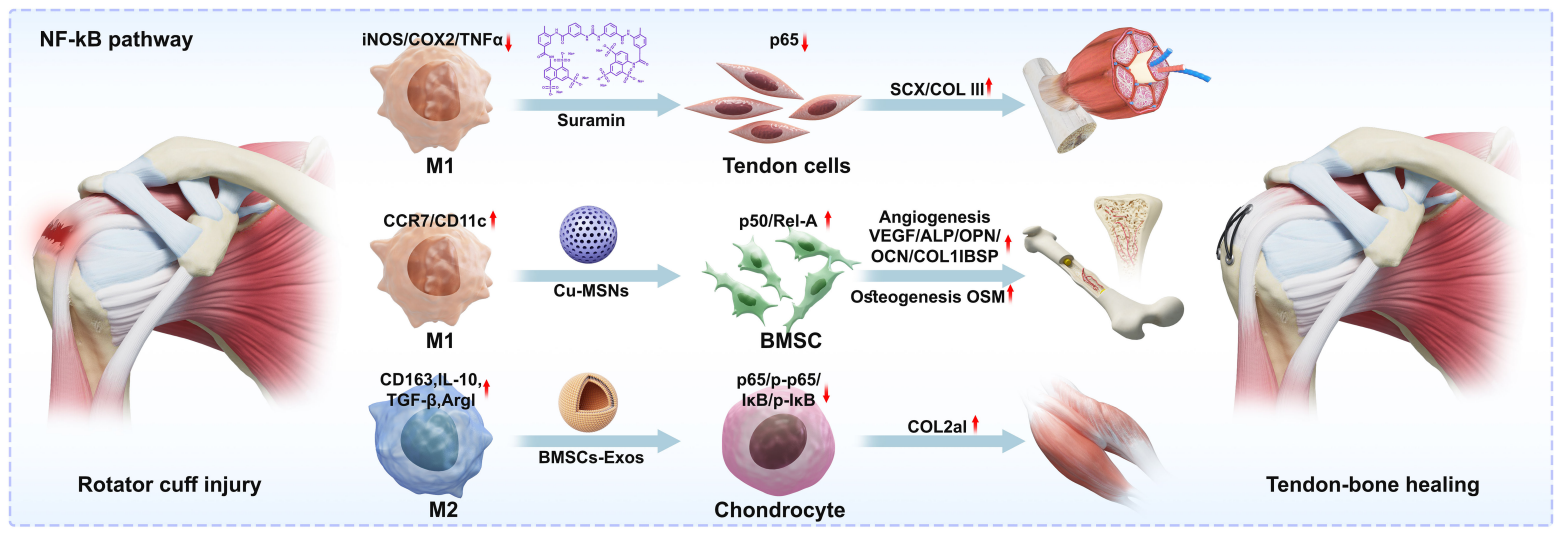
Tendon bone healing factors linked to the traditional NF-kB pathway that macrophages support. Exos act on proteins upstream or downstream of the regulatory pathway in tendon cells, mesenchymal stem cells, chondrocytes, etc., respectively. These proteins then regulate osteogenesis, vasculogenesis, or tendonogenesis, which ultimately aid in tendon-bone healing. Macrophages polarised to M1 or M2 in response to factors like suramin, Cu-MSNs, and BMSCs.

Bioactive ions such as copper (Cu) can mimic hypoxic environments by regulating hypoxia-inducible factor (HIF) to upregulate downstream angiogenesis-related genes such as VEGF (21). Copper-doped mesoporous silica nanospheres (Cu-MSNs) were synthesised under near-neutral conditions (pH=6) using ammonium fluoride as a catalyst, through the synergistic self-assembly of copper salts and the silica precursor (TEOS), enabling uniform incorporation of Cu²^+^ into the silica network. As shown in **Figure 2**, Following uptake of Cu-MSNs by macrophages (Mφ), multistage signalling pathways regulate tendon-bone healing: firstly, Cu²^+^-mediated hypoxia simulation markedly reduces intracellular reactive oxygen species (ROS) levels, mitigating inflammatory injury; secondly, TLR3/4 receptors are activated, triggering the NF-κB pathway via Ticam1/2 to induce M1 polarisation; Subsequently, M1-polarised macrophages secrete oncolysin M (OSM), activating the OSM–OSMR/IL6ST–STAT3 axis to promote osteogenic gene expression in BMSCs while simultaneously upregulating OPG and suppressing RANKL, thereby generating a dual effect of ‘promoting osteogenesis while inhibiting osteoclastogenesis’. Additionally, Cu²^+^ and Si^4+^ synergistically promote angiogenesis, alleviating blood supply insufficiency in large bone defect areas. This study achieves inorganic nanomaterial-mediated bone-immune regulation and integrated tendon-bone interface regeneration through immune microenvironment reprogramming, offering novel therapeutic insights (29).

Electroactive biomaterials (EABs) modulating the cellular microenvironment represent a recent research focus. EABs transcend mere conductive materials, functioning as bioelectronic scaffolds with extensive biointeraction capabilities. They dynamically sense tissue signals, interact with tissue cells, and regulate the electrophysiological microenvironment (30, 31). MXene, as an electroactive biomaterial, has also demonstrated exceptional regulatory capacity over macrophages. Research has confirmed that intracellularly internalised transition metal carbide/nitride (MXene) nanosheets serve as the sole regulatory factor in modulating macrophage polarisation (32). Internalised MXene nanosheets utilise their high electrical conductivity and magnetoelectric properties to generate electrical signals and reactive oxygen species (ROS) under rotating magnetic fields, thereby inducing M1 macrophage polarisation. Upon removal of the magnetic field, the bioactivity of MXene nanosheets promotes the repolarisation of macrophages towards the M2 phenotype. The regulatory mechanism of these nanosheets involves inhibiting the nuclear factor κB (NF-κB) signalling pathway activated by B cells, alongside activating the Janus kinase-signal transducer and activator of transcription (JAK-STAT) pathway. Under switched rotating magnetic field stimulation, MXene nanosheets achieve continuous macrophage polarisation from M1 to M2, effectively promoting tissue regeneration.

Beyond regulating damage to normal tendon-bone tissue, modulating pathological microenvironments such as those in bone tumours and fibrotic tendon-bone healing surfaces may also offer novel insights. One research team developed thermosensitive liposomal nanoparticles loaded with rimovert to promote M1 macrophage polarisation, thereby regulating the immune microenvironment and enhancing tissue regeneration (33). R848 promotes M1-like macrophage polarisation and serves as an effective immunostimulatory agent in cancer immunotherapy. Another research group synthesised SKCN, a novel quinolone-based fully fused ring receptor-donor-receptor (A-D-A) molecule, and prepared FA-SNPs and FA-SRNPs nanoparticles. By incorporating the immunomodulator R848, FA-SRNPs effectively modulate the tumour immune microenvironment, reducing Treg and M-MDSC infiltration while promoting dendritic cell maturation and M1 macrophage polarisation. This maturation of DCs and M1 macrophage polarisation effectively activates cytotoxic T lymphocytes within tumours (34). An alternative delivery system based on TAM-targeting albumin nanoparticles (M@SINPs) was employed for co-delivery of photosensitiser IR820 and SHP2 inhibitor SHP099 to enhance macrophage-mediated cancer immunotherapy (35). Under laser irradiation, M@SINPs generate intracellular reactive oxygen species (ROS), promoting the shift of M2-TAMs towards an M1 phenotype. Concurrently, SHP2 inhibition blocks the CD47-SIRPa pathway, restoring phagocytic activity in M1 macrophages. This repolarises TAMs towards the M1 phenotype, reactivating phagocytosis, promoting tumour-infiltrating CTLs, and generating an immunostimulatory tumour microenvironment (TME). Consequently, tumour growth is significantly suppressed while normal tissue growth is facilitated.

Silicon dioxide nanoparticles (SiNPs) are widely utilised engineered nanomaterials (36–38). SiNPs drive RAW264.7 cell polarisation towards the M1 phenotype via H3K18 lactylation (H3K18la), concurrently increasing IL-6 and TNF-α secretion. This SIRT3/H3K18la/NOS2 axis establishes a novel pathway driving macrophage M1 polarisation within the fibrotic microenvironment, positioning SIRT3 as a promising therapeutic target for intervening in SiNP-induced fibrosis (39).

### 4.2 Chemotactic factor

CCL4L2 is a chemokine that recruits macrophages to sites of injury and initiates the inflammatory response (40). It promotes M1 polarisation of macrophages by activating the TNF-α receptor and NF-κB signalling pathway, thereby enhancing pro-inflammatory responses. Following M1 polarisation, CCL4L2 upregulates genes such as SPP1, promoting endochondral ossification and tissue repair. Concurrently, CCL4L2 inhibits the differentiation of tendon stem/progenitor cells (TSPCs) into other immune cell types, thereby constraining excessive inflammation and maintaining local immune homeostasis (41). Collectively, CCL4L2 exhibits dual regulatory functions: promoting M1 polarisation to enhance defensive responses during early inflammation, while suppressing abnormal TSPC differentiation to prevent inflammatory excess in later stages, thus mediating a ‘pro-inflammatory initiation–repair equilibrium’ (42).

CXCL12 is the star factor in tendon-bone healing research. It recruits not only macrophages but, more importantly, bone marrow-derived mesenchymal stem cells (BMSCs) via the CXCR4 receptor. MSCs are pluripotent stem cells involved in the differentiation processes of bone, cartilage, adipose tissue, tendons, and muscle. MSCs are recruited to target sites via microenvironmental chemical signals, including CXCL12 and its receptor CXCR4. CXCL12 influences MSC migration, growth, survival, and differentiation (43). The CXCL12/CXCR4 axis plays a crucial role in myoblast migration and regeneration (44). Regulation of macrophage M1 polarisation via this axis subsequently influences BMSC and myoblast differentiation, thereby promoting tendon-bone union.

CCL2/CCR2 enhances NMDA receptor function by activating the ERK-GluN2B pathway, promoting synaptic long-term potentiation (LTP) to enhance synaptic plasticity. It also drives M1 polarisation of microglia and activation of astrocytes, releasing substantial inflammatory mediators (e.g., TNF-α, IL-1β, etc.) and induces astrocytes to produce more CCL2, thereby forming a ‘neuron-glia’ positive feedback network. Furthermore, this pathway also causes a ‘disinhibition’ effect by suppressing GABAergic interneuron function, thereby removing restrictions on pain pathways, and promotes opioid tolerance by inducing μ-opioid receptor heterodimer desensitisation, reducing the analgesic efficacy of opioid drugs, collectively maintaining central sensitisation (45). Persistent chronic pain and the release of numerous inflammatory mediators by M1-polarised macrophages promote extensive M2 generation. This alleviates the inflammatory effects of M1 macrophages, prevents excessive tissue damage, and facilitates the repair of bodily injury.

Beyond CCL4L2, CXCL12, and CCL2, numerous chemokines such as CXCL10 (IP-10), CXCL9 (MIG), and CCL5 (RANTES) participate in the M1 polarisation process. Studies have also suggested that MIP-1 and CXCR3, as chemokines, may be key molecules potentially regulating macrophage M1 polarisation to promote tendon-bone healing, though these are not elaborated upon here. Beyond chemokines, numerous signalling molecules such as PFKFB3 and PlexinD1 exert influence over the macrophage-dominated immune microenvironment, warranting further investigation and discussion.

In summary, materials promoting M1 macrophage polarisation during the early inflammatory phase predominantly function by accelerating the destructive stage of early inflammation or by inducing the expression of tissue regeneration factors through molecular regulation. The NF-κB pathway, frequently cited in this context, represents a pivotal regulatory pathway for M1 polarisation during early inflammation and warrants prioritisation in subsequent investigations into macrophage immune regulation. Nanomaterials, a recent focal point in materials science research, demonstrate considerable promise—from the initial trend of drug nanomaterialisation to material nanomaterialisation, and encompassing nanostructures, nanoparticles, and nano-EABs. Numerous nanomaterials can simultaneously regulate macrophage polarisation and carry nanodrugs for dual modulation. Incorporating pH-responsive chemical bonds like Schiff bases, or other temperature-, ultrasound-, current-, or light-responsive bonds, enables corresponding conditional or sequential regulation. Beyond regulating normal tissues, immunological research targeting pathological fibrosis at bone tumour or healing tendon surfaces is equally essential. M1 macrophages function as ‘nuclear weapons’ delivering precision-guided strikes against abnormal tissue, promoting its clearance or retarding fibrosis progression to cultivate favourable conditions for normal tissue growth. However, we must clearly recognise that macrophage polarisation is a continuous process. Consequently, various chemokines regulating the immune processes in tendon-bone healing often promote M1 polarisation followed by a sequential transition towards M2, thereby further optimising the immune microenvironment. By targeting materials as entry points for macrophage regulation, employing temporal control as a ‘remote control’ for inflammatory progression and regenerative repair, and conducting in-depth mechanistic research to grasp the ‘steering wheel’ of immune regulation, we believe macrophage-mediated immune control will emerge as a formidable weapon in our arsenal against numerous diseases.

## 5 Factors and mechanisms inhibiting M1 macrophage polarisation in late-stage inflammation to promote tendon healing

### 5.1 Small extracellular vesicles

Adipose-derived stem cells (ADSCs) secrete small extracellular vesicles that have been demonstrated to promote bone regeneration by regulating macrophage polarisation (46). Compared to MSCs isolated from bone marrow or umbilical cord, ADSCs can be readily obtained from abdominal fat aspiration in patients, offering a more convenient and less invasive source for acquiring small extracellular vesicles (47). Consequently, small extracellular vesicles (sEVs) derived from ADSCs represent an optimal choice for encapsulating diverse hydrogel materials and drug delivery system carriers.

Circadian rhythms, which have long been acknowledged as important in physiology, are now thought to be important modulators of particular immunological activities, affecting the release of inflammatory cytokines (48, 49). The inflammatory environment mediated by Macrophages is essential for TBI recovery. Excessive M1 Macrophages pro-inflammatory molecules inhibit stem cell chondrogenic development, which prevents the regeneration of the fibrocartilage layer (50, 51). By delivering platelet-derived growth factor 4 (PF4), which increases cyclic adenosine monophosphate (cAMP) signalling in Macrophages and prevents their polarisation toward the pro-inflammatory M1 phenotype, circadian rhythm-regulated small extracellular vesicles (CR-sEVs) modify the inflammatory microenvironment. This anti-inflammatory action enhances tendon-bone junction repair and encourages fibrocartilage regeneration. Additionally, tendon decellularised extracellular matrix (T-dECM), which was integrated into the microneedle base, improved tendon repair when CR-sEVs loaded onto triphasic microneedle (MN) scaffolds were locally supplied to the injury site. In a rat rotator cuff repair (RCR) model, CR-sEVs work in concert with MNs to suppress inflammation and promote tissue regeneration, which greatly improves biomechanical strength and shoulder joint function and leads to effective tendon-to-bone healing (52, 53). A research team has recently developed a novel macroporous hydrogel—sodium alginate/hyaluronic acid/mechano-hormone-activated stem cell-derived extracellular vesicles (MHA-sEVs). The microfibrillar gel arrangement within MHA-sEVs induces tendon-derived stem cells towards tendon differentiation, whilst the sEVs themselves ameliorate mitochondrial dysfunction in M1-type macrophages (Mφ) and inhibit Mφ polarisation towards the M1 phenotype via the nuclear factor-κB (NF-κB) signalling pathway (53).

### 5.2 Electrospinning

Electrospinning has emerged as a prominent research focus in recent years. As an emerging material exhibiting high mechanical strength, not only provides mechanical properties but also serves as a drug delivery system capable of loading multiple functional drugs. Alternatively, the material’s inherent structure can provide temporal response conditions for immune regulation. For instance, using sericin as the spinning substrate and modifying its surface with octadecylamine via Schiff base bonds yields pH responsiveness. At low pH, the Schiff base bonds break, releasing the drug-loaded system linked to octadecylamine. Materials combining electrospinning with delivery systems to modulate macrophage-dominated immune microenvironments have proliferated in recent years.

One research team developed novel strontium-doped mesoporous bio-glass nanospheres (Sr-MBG), which were electrospun onto nanofibre scaffolds to successfully produce electrospun fibre scaffolds (BIIEFS) with dual-system induction and immune-modulating capabilities. BIIEFS continuously release bioactive ions, including strontium (Sr), calcium (Ca), and silicon (Si). It promotes osteogenic and chondrogenic differentiation of bone marrow-derived mesenchymal stem cells via the Wnt pathway while inhibiting macrophage M1-type polarisation (51). The integration of electroactive biomaterials with electrospinning represents another contemporary research focus. Surgical sutures engineered with electroactive, anti-inflammatory properties—featuring a core of high-strength polylactic acid (PLA) micron yarns and a PEDOT shell—effectively promote tendon-bone regeneration in Achilles tendon avulsion injuries. Electrical stimulation enhances tendinogenic and osteogenic differentiation by activating cellular signalling pathways such as PI3K/Akt, while CS modulates NF-κB and BMP-2 pathways via chemical signals to inhibit M1 polarisation, promote anti-inflammation, and stimulate regeneration. This synergistic approach establishes an ‘electro-chemical dual-regulation’ repair microenvironment, significantly enhancing repair efficiency (54). Additionally, a research team utilised electrospinning technology to produce a nanofibre composite (SF/PHBV) from sericin (SF) and poly(3-hydroxybutyrate-co-3-hydroxyvalerate) (PHBV). Berberine (BBR) was then loaded onto this SF/PHBV matrix to fabricate a nanofibre wound dressing (SF/PHBV-BBR) (55). Berberine inhibits the TGF-β/Smad signalling pathway by downregulating p-Smad3, thereby suppressing M1 macrophage polarisation and fibrosis at the tendon-bone healing interface. This promotes tendon-bone union while reducing scar formation at the healing site.

In the late stages of inflammation, immune regulation is predominantly mediated by the M2 macrophage phenotype. Consequently, the M1 phenotype, which promotes the release of inflammatory cytokines, should be suppressed to facilitate inflammatory resolution and tendon-bone healing processes. In recent years, numerous pseudo-decorated delivery systems utilising biomaterials such as small extracellular vesicles, liposomes, and macrophage membranes as encapsulation agents have emerged, all yielding satisfactory outcomes. The rationale lies in utilising these cell membranes as ‘targeted radars’ for delivery to homologous cells or sites requiring precise intervention. Concurrently, external environmental factors such as circadian rhythms play a crucial role in regulating the immune microenvironment. The integration of these approaches represents a fusion of targeted delivery with environmental responsiveness. Electrospinning, as an emerging carrier technology, offers comprehensive responsive capabilities, including pH-responsive, ultrasound-responsive, and photothermal-responsive properties. Integrating electrospinning with the aforementioned approaches, utilising materials as a substrate and incorporating multifunctionalisation, holds immense potential for immunomodulatory tendon-bone healing.

## 6 Factors and mechanisms promoting M2 macrophage polarisation to facilitate tendon-bone healing in the late inflammatory phase

### 6.1 Various scaffold materials

#### 6.1.1 3D-printed polycaprolactone scaffolds loaded with bFGF and BMSCs

In recent years, natural polymers, synthetic polymers, and composite materials have been extensively employed in tissue engineering research for tendon-bone healing (56). Multilayer scaffolds can mimic the complex structure of the tendon-bone interface, providing an optimal microenvironment for cellular infiltration and growth, thereby enhancing biocompatibility and promoting tissue integration (57, 58). Among these, polycaprolactone (PCL) has emerged as an ideal scaffold material due to its excellent biocompatibility, controllable degradability, and mechanical properties matching those of tendon and bone tissues (59, 60). Three-dimensionally printed PCL scaffolds loaded with bFGF and BMSCs (PCLMF) modulate macrophage gene expression by downregulating pro-inflammatory genes and upregulating anti-inflammatory genes, thereby promoting M1-to-M2 polarisation to suppress inflammation and facilitate repair. This effect is closely associated with the inhibition of the MAPK signalling pathway. BMSCs further promote M2 polarisation by modulating the IL-10/STAT3 pathway, synergising with the immunomodulatory effects of PCLMF to significantly enhance tendon-bone interface repair capacity.

#### 6.1.2 Wnt3a scaffolds modulate macrophages for improved TBI healing

**Figure 3:**
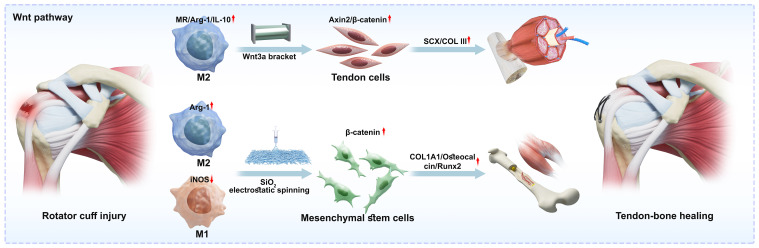
Elements linked to the traditional Wnt pathway, where macrophages aid in the mending of tendon bones. In response to stimuli like Wnt3a brackets and SiO2 electrostatic spinning, macrophages polarise to M1 or M2. They then interact with proteins upstream or downstream of the regulatory pathway, such as mesenchymal stem cells and tendon cells, respectively, to control osteogenesis, vasculogenesis, or ten.

As illustrated in **Figure 3**, the Wnt3a-modified bionic scaffold remodels the immune microenvironment through sustained release of Wnt3a protein, enhancing M2 polarisation to promote tendon regeneration. The mechanisms comprise: (1) Activation of the classical Wnt/β-catenin signalling pathway: As illustrated in **Figure 2**, Wnt3a acts as a key ligand that specifically binds to the LRP5/6 receptor, simultaneously interacting with the seven-transmembrane receptor Frizzled (Fzd) and the single-transmembrane co-receptor LRP5/6 on the cell membrane. Formation of this ternary complex (Wnt-Fzd-LRP6) leads to phosphorylation of the LRP6 intracellular domain, thereby recruiting the cytoplasmic scaffolding protein Dishevelled (Dvl). In the absence of Wnt3a, cytoplasmic β-catenin is captured and degraded by a ‘destabilising complex’. This complex comprises Axin, APC, GSK-3β, and CK1. GSK-3β phosphorylates β-catenin, leading to its ubiquitination and degradation. Upon recruitment to the membrane, Dvl inhibits GSK-3β activity, causing the disruptor complex to disassemble. This prevents β-catenin phosphorylation and degradation, thereby activating its transcriptional activity to regulate monocyte differentiation towards the M2 phenotype. Stabilised β-catenin accumulates in the cytoplasm and translocates to the nucleus. Within the nucleus, β-catenin displaces transcriptional repressors, binds to transcription factors of the TCF/LEF family, and initiates downstream gene transcription. This includes key transcription factors promoting M2 macrophage polarisation, such as Irf4 and Jmjd3; (2) Polarisation synergistic amplification effect: IL-4 in the microenvironment binds to the IL-4Rα receptor on the macrophage surface, activating JAK1/JAK3 kinases, which in turn lead to the phosphorylation and dimerisation of STAT6. Nuclearised β-catenin can act as a co-activator for STAT6. β-catenin and STAT6 proteins form complexes within the promoter regions of M2 marker genes. This interaction may recruit histone acetyltransferases, altering chromatin structure to enhance transcription accessibility for M2-associated genes. Under IL-4 microenvironmental stimulation, Wnt3a reinforces the M2 polarisation state by upregulating Arg-1 and CD206 expression, thereby establishing a pro-regenerative molecular phenotype; (3) Paracrine signalling: M2 macrophages secrete factors such as VEGF and BMP-2, which stimulate osteogenic differentiation of tendon stem cells through Wnt–Notch signal interaction, thereby promoting fibrocartilage reconstruction. This scaffold achieves precise regulation of inflammatory and regenerative signals (61).

#### 6.1.3 MFG-E8 scaffolds promote efferocytosis and M2 transition

Milk fat globule-derived epidermal growth factor-8 (MFG-E8) and its recombinant protein (rMFG-E8) modulate macrophage phagocytic function and polarisation status via the efferocytosis pathway, thereby promoting tendon healing. The mechanisms involved include: (1) enhancing macrophage recognition of the ‘eat me’ signal from apoptotic cells, clearing apoptotic cells and promoting M2 polarisation; (2) Inhibiting TLR4 signalling via binding to integrin αvβ3, partially reversing iNOS expression in M1 macrophages; (3) Enhancing CD206 expression and reducing iNOS expression at the early interface stage, thereby mitigating inflammation and promoting osseointegration. This study reveals MFG-E8’s bidirectional signalling for comprehensive anti-inflammatory and pro-regenerative regulation, providing novel immunotherapeutic targets for tendon healing (62, 63).

#### 6.1.4 Chitosan-loaded scaffolds in the joint-tendon-bone

The chitosan scaffold loaded within the joint-mimetic adhesive layer (JTA) promotes gradient healing of traumatic brain injury (TBI) by remodelling the immune microenvironment through modulating macrophage (Mφ) phenotype. The deacetylation activation effect of positive charges on the chitosan surface drives M1-to-M2 polarisation, upregulating expression of CD206 and factors including IL-10 and TGF-β. M2 macrophages promote angiogenesis and collagen remodelling through a VEGF–BMP-2 signalling cascade, enabling interface regeneration driven by the ‘macrophage–stem cell coupling’. This strategy reveals a novel direction for designing gradient-integrated biomaterials based on immune homeostasis regulation (64).

#### 6.1.5 Topo-engineered scaffolds programme macrophage phenotypes via physical signals

Decellularised extracellular matrix (aECM) scaffolds with ordered microchannel structures regulate regeneration through biophysical signals, primarily via three mechanisms: (1) Topography-dependent polarisation: The RhoA/ROCK pathway mediates morphological stretching of macrophages, triggering YAP/TAZ nuclear translocation and upregulating Arg-1/IL-10 expression; (2) Mechanical transduction regulation: 3% cyclic stretching promotes Ca²^+^ influx via the PIEZO1 channel, inhibiting NFAT5-driven IL-1β/iNOS expression; (3) Paracrine network remodelling: polarised M2 macrophages secrete PDGF-AA/TGF-β1 to recruit tendon progenitor cells, facilitating ordered ECM deposition (65).

#### 6.1.6 BMnPs-functionalised scaffolds

Bismuth-magnesium hydroxyapatite particles (BMnPs) can specifically induce Mφ to polarise towards the M2 phenotype, activating the transcription factor cMaf to upregulate IL-10 expression, thereby forming an anti-inflammatory–pro-osteogenic synergistic effect via both autocrine and paracrine mechanisms. PGE2 released by MSCs binds to macrophage EP2/EP4 receptors, forming a positive feedback loop that further amplifies IL-10 signalling. Compared to conventional micron-scale hydroxyapatite, BMnPs scaffolds not only reduce inflammatory responses by promoting M2 polarisation but also stimulate the release of pro-angiogenic factors, accelerating bone regeneration and mineralisation through both mechanical and molecular mechanisms. This mechanism reveals the pivotal role of immunomodulatory materials in osseointegration (66, 67).

### 6.2 Other materials

#### 6.2.1 Mg²^+^

Biodegradable magnesium-based materials promote tendon-bone interface healing through dual mechanisms of immunomodulation and tissue regeneration by releasing Mg²^+^. Research indicates that Mg²^+^ significantly enhances levels of osteogenesis-related factors in periosteal-derived stem cells (10). At the immunological level, Mg²^+^ activates the STAT6 signalling pathway to drive Mφ towards M2 phenotype polarisation, upregulating CD206/Arg expression while suppressing TLR/NF-κB-mediated TNF-α and IL-6 secretion. This shapes an anti-inflammatory microenvironment and enhances alkaline phosphatase activity in mesenchymal stem cells. At the regenerative level, Mg²^+^ promotes BMSC adhesion and osteogenic differentiation via the α5β1 integrin/FAK pathway. Furthermore, it activates the Hif-1α/Sox9 cascade through TRPM7 channels, inducing Acan and Col II synthesis to achieve chondro-like tissue reconstruction. This synergistic ‘immune amplification-signal decoding’ network increases new bone formation by 58%, providing a multi-target intervention strategy for TBI repair (68, 69). Furthermore, porous magnesium or surface-etched structures can similarly induce Mφ to polarise towards the M2 phenotype, accelerating tendon-bone healing (70–74). Magnesium-based materials are biodegradable and are completely metabolised and excreted, gradually disappearing as tissue regenerates, thereby further promoting repair (75–79). Demonstrating the potential of magnesium-based materials and magnesium-pretreated periosteum in tendon healing research (80).

High-purity magnesium screws (HP Mg) exert precise immune regulation via the Mg²^+^ released through the AKT1/PI3K pathway: (1) Mg²^+^ inhibits AKT2 phosphorylation and enhances AKT1 activation via the TRPM7 channel, inducing MRC1/CD163 expression mediated by STAT6; (2) M2 macrophages secrete PDGF-BB/TGF-β3 to activate the SOX9/RUNX2 pathway in hBMSCs, promoting synthesis of type II collagen and aggrecan; (3) At 12 weeks post-surgery, interfacial tensile strength reached (32.5 ± 3.1) MPa, comparable to autologous grafts, achieving conversion from fibrovascular scar to functional fibrocartilage (81, 82).

#### 6.2.2 TiO_2_ honeycomb topology

For the promotion of osseointegration, micro-surface topography is essential when the bone-immune tissue microenvironment is suitably adjusted (83–86). Additionally, when cells are stretched in particular ways, microsurface topography can cause M2 macrophage polarisation (87–89). The construction of TiO_2_ honeycomb topologies spanning a gradient scale (90–5000 nm) reveals a mechanism whereby nanomorphology modulates Mφ polarisation through mechanical signal transduction. Nanostructures HC-90, fabricated via thin-film transfer, significantly enhanced Mφ pseudopod formation and promoted spatial morphoplasticity, thereby inducing M2 polarisation and secretion of IL-4, IL-10, and BMP-2 to establish a pro-regenerative immune microenvironment. Mechanistic studies indicate the RhoA/ROCK pathway plays a central role in nanotopology-mediated immune regulation: HC-90 upregulates cytoskeletal genes, including RhoA and Arp2/3, while suppressing TNF-α and NF-κB signalling. Concurrently, elevated p-MLC/p-MYPT1 phosphorylation enhances actin contractility, promoting footplate formation. Furthermore, PPARγ signalling activation and MAPK inhibition jointly drive M1-to-M2 macrophage conversion. This immunoregulatory effect significantly enhances mesenchymal stem cell alkaline phosphatase activity and mineralization capacity in *in vitro* co-culture models, while *in vivo* experiments confirm its ability to increase new bone volume fraction and bone-implant contact rate. This study proposes an integrated mechanism of ‘nanoscale confinement-mechanical transduction-immune regulation’, providing theoretical foundations and design principles for surface topography-engineered immune-modulatory bone implant materials (74).

### 6.3 Biological factors: adipose-derived stem cells

Because they control macrophage polarisation and alter the milieu, adipose-derived stem cells (ASCs) are essential for tendon-bone repair. According to research, Macrophages can be made to differentiate into the M2 anti-inflammatory phenotype by ASCs. Significant overexpression of its hallmark markers, IL-4 and CD163, can inhibit the TNF-α-mediated pro-inflammatory cascade. One cytokine linked to tendon inflammation, degeneration, and apoptosis is TNF-α, which also helps to prevent tendon-derived stem cells (TDSCs) from proliferating (90). By functionally connecting with T lymphocytes, endothelial cells, and fibroblast precursor cells, M2 macrophages create a regenerative microenvironment. Their secreted extracellular matrix remodelling proteins work in concert to increase the ability of fibroblasts to proliferate and synthesize collagen. ASCs upregulate matrix metalloproteinases, which speed up the structural remodelling of functional tendon matrix, and activate the endothelial growth factor signalling pathway, which further promotes neovascularisation and basal cell migration, especially in the BMP-12 co-treatment system. This multifaceted regulatory mechanism facilitates a seamless transition of the tendon-bone regeneration process from the inflammatory phase to the proliferative phase by efficiently suppressing prolonged inflammation brought on by monocyte infiltration. In the end, thorough optimization of the cell-matrix-immune network results in high-quality tendon-bone regeneration (91).

### 6.4 Drug: baicalin

Baicalin (BCL) is an active component extracted from the traditional Chinese medicinal herb Scutellaria baicalensis, which has been demonstrated to possess anti-inflammatory, antioxidant, anti-cancer and anti-diabetic functions, and is widely utilized for various diseases. Baicalin (BCL) promotes tendon-bone healing by regulating macrophage polarisation. Based on a dual-targeted nanodelivery system (BCL@MMSNPs-SS-CD-NW), BCL inhibits ROS/NF-κB signalling pathways to induce M2-type macrophage polarisation, thereby driving mesenchymal stem cell (MSC) osteogenic differentiation and accelerating bone repair. Research indicates that BCL dose-dependently inhibits H_2_O_2_-induced ROS accumulation, blocks NIK activation in the non-canonical NF-κB pathway, reduces p-p65 and p-IκB levels, suppresses NF-κB nuclear translocation and pro-inflammatory factor release, thereby promoting the M1-to-M2 macrophage transition. Co-culturing M2-type macrophages with MSCs significantly enhances MSC osteogenic differentiation, increasing COL-I, OCN, and Runx2 expression while elevating ALP activity and mineralization levels. This drug delivery system integrates a Fe_3_O_4_ magnetic core with macrophage-targeting peptide NW, enabling targeted aggregation under an external magnetic field. Intracellular GSH triggers BCL release, achieving precise immune regulation. *In vivo* experiments demonstrated that this system markedly enhances local BCL bioavailability and M2 macrophage infiltration, accelerating callus maturation and increasing bone density. This effect relies on a synergistic mechanism involving ROS/NF-κB pathway inhibition and MSC osteogenic differentiation. This research provides novel insights for targeted immunomodulatory bone regeneration therapies (92).

In macrophage-dominated immune regulation, M2 macrophages occupy an absolutely pivotal position, as they govern the entire healing process. Consequently, numerous materials target M2 macrophages as their regulatory focus. The aforementioned scaffold materials, metal ions, surface topography, and various pharmaceuticals each modulate M2 macrophage polarisation from distinct angles. However, designing an entirely novel material system that integrates these approaches may represent the ultimate strategy for regulating M2 macrophage polarisation. Building upon this concept, we propose a metal-based drug delivery system utilising metal-organic framework (MOF) scaffolds with topologically etched micro-topography. The metallic material should exhibit degradability or the capacity to release metal ions. Elements such as magnesium (Mg) and silver (Ag) have been demonstrated to regulate wound healing and bone tissue regeneration, serving as fundamental modulators of tendon-bone union. Surface microtopographies featuring hexagonal or spindle-shaped structures have also been shown to enhance cell adhesion, thereby promoting vascular regeneration and macrophage aggregation. Specific geometries can further facilitate macrophage differentiation towards the M2 phenotype. Moreover, numerous drugs targeting macrophage M2 polarisation exist, with the challenge lying in their nanoscale formulation and encapsulation within suitable metallic MOFs for precise release at specific times or locations. However, with advances in materials science and molecular biology, these obstacles are likely to be overcome. Integrating multiple materials based on the aforementioned approach may offer novel insights into regulating the macrophage-dominated immune microenvironment.

## 7 Mechanisms by which inhibition of M1 and promotion of M2 macrophage polarisation enhance tendon-bone healing in late-stage inflammation

### 7.1 Environmental factors

#### 7.1.1 High ROS environment

Reactive oxygen species (ROS) encompass superoxide anion (O_2_^-^), hydroxyl radical (HO_2_), peroxyacetyl radical (RO_2_), alkoxy radicals, and non-radical compounds readily converted into radicals such as hydrogen peroxide (H_2_O_2_) and hypochlorous acid (HOCl), which may impede tendon-to-bone healing (93). As normal by-products of oxygen metabolism, ROS can function as signalling molecules to rapidly respond to stimuli during acute inflammation, whilst also potentially triggering oxidative stress (94, 95). Research indicates that reactive oxygen species (ROS) promote M2 polarisation via the ROS/PI3K/Akt pathway or H_2_O_2_-mediated mechanisms, whilst antioxidants suppressing endogenous ROS reduce M2 marker expression. Part of this effect is mediated through the inactivation of Stat3 during IL-4-induced M2 polarisation (96). Overall, high ROS environments tend to induce M1 polarisation, whereas moderate or low ROS levels favour M2 polarisation, suggesting that ROS exerts a bidirectional regulatory role in immune modulation and tendon repair (94).

ROS has been demonstrated to be one of the most critical factors inducing inflammatory states, capable of rapidly differentiating macrophages into the M1 phenotype, recruiting substantial production of inflammatory cytokines, and triggering neuropathic responses at injury sites, thereby causing persistent pain. The scavenging of ROS remains a prominent research focus, with numerous ROS-targeting therapeutics such as Prussian blue nanozymes now widely employed. However, research into ROS should not be confined solely to its role as an inflammatory trigger; it must also be recognised as an integral component of the inflammatory microenvironment. Regulation of the immune microenvironment should therefore focus on holistic environmental control. Providing optimal ‘soil conditions’ for ‘seed cells’ such as BMSCs and HUVECs during tendon healing is essential to promote their healthy proliferation, thereby achieving near-complete, highly efficient repair at the tendon interface.

#### 7.1.2 Biomechanical and topographic cues synergize to modulate macrophage–fibroblast interactions

In orthopaedic surgery, natural or synthetic biomaterial patches are widely employed to enhance the strength of soft tissue repair (97–100). Surface topography can induce M0 macrophages to polarise towards the M2 phenotype while suppressing excessive pro-inflammatory cytokine secretion, whereas randomized fibrillar structures combined with dynamic mechanical stretching promote M1 polarisation. Mechanically stimulated Mφ induce fibroblast pro-inflammatory responses via NF-κB-p65 nuclear translocation; however, pre-mechanical stimulation confers ‘mechanical protection’ to fibroblasts through the Rho/ROCK pathway, attenuating IL-1β-induced inflammatory cascades. Dynamic mechanical loading interacts with fibroblasts to significantly reduce the pro-inflammatory CCR7^+^ subpopulation while activating the MRC1^+^ M2 phenotype, suppressing NF-κB overexpression and establishing an anti-inflammatory microenvironment (101).

Appropriate mechanical loading remodels the immune microenvironment through multi-level signalling: it inhibits the TLR4/NF-κB pathway in macrophages while activating the IL-4/JAK1/STAT6-PPARγ repair axis, thereby promoting M2 polarisation and enhancing TGF-β1 secretion. Concentration-dependent activation of the SMAD2/3 pathway in mesenchymal stem cells (MSCs) by TGF-β1 released from M2 macrophages induces fibrocartilage cell differentiation. STAT6-PPARγ and YAP/TAZ signalling synergistically regulate TGF-β1 biosynthesis and spatial gradients; excessive mechanical loading reverses repair via NLRP3 inflammasome activation (102, 103). *In vivo* experiments demonstrate that specific depletion of macrophages completely abolishes the wound-healing-promoting effect of mechanical stimulation, resulting in reduced fibrocartilage layer thickness. Furthermore, early postoperative immobilization diminishes M1 macrophage accumulation, alleviates acute inflammation, and enhances the continuity of fibrous scar tissue and collagen (104). Overall, the biomaterial’s topology and mechanical loading precisely modulate the interaction between monocytes/macrophages and fibroblasts, thereby optimizing healing at the tendon-bone interface.

### 7.2 Scaffold-related materials

#### 7.2.1 Lithium-containing mesoporous silica

Mesoporous bioactive glass can modulate the immune microenvironment by incorporating bioactive components (105, 106). During degradation, Li-MSN scaffolds continuously release lithium and silicon ions. Lithium activates the Wnt/β-catenin pathway to promote osteogenic differentiation at the tendon-bone interface, while synergistically inducing the transition of macrophages from pro-inflammatory M1 to anti-inflammatory M2 cells, thereby suppressing local inflammation. M2 macrophages accelerate regeneration by secreting anti-inflammatory and angiogenic factors. Furthermore, silicon ions inhibit RANKL-mediated osteoclast activation, thereby creating a favourable environment for bone regeneration (51).

#### 7.2.2 Scaffold loaded with TSPC-derived exosomes (Col@PDA&T-Exos)

The Col@PDA&T-Exos scaffold activates important fibrogenic factors and modifies macrophage polarisation to dramatically improve rotator cuff tendon-to-bone repair. According to *in vitro* tests, the TSPC-Exos scaffold created a beneficial anti-inflammatory milieu for healing by inhibiting M1 macrophage polarisation and encouraging M2 macrophage migration. Additionally, *in vivo* research shows that the TSPC-Exos scaffold promotes the distribution of M2 Macrophages near the tendon-bone interface in the early postoperative phase and inhibits M1 Macrophages’ activity, which speeds up the healing process. Furthermore, miRNA-Seq research shows that the TSPC-Exos scaffold releases miR-21a-5p, which may target the PDCD4/AKT/mTOR signalling pathway and induce fibrosis. In particular, miR-21a-5p probably inhibits the production of PDCD4, which triggers the AKT/mTOR signalling pathway and encourages BMSC migration, proliferation, and fibrotic differentiation. In addition to demonstrating the TSPC-Exos scaffold’s possible function in controlling macrophage polarisation, this method offers essential theoretical justification for comprehending its possible uses in regenerative medicine (107).

#### 7.2.3 Bionic delivery system constructed from M2Macrophages and polylactic-polyhydroxyacetic acid (PLGA) nanoparticles (M2M@PLGA/COX-siRNA)

The M2M@PLGA/COX-siRNA biomimetic delivery system employs the homotargeted properties of M2Mφ membranes to achieve precise enrichment at injury sites. The PLGA core enables controlled intracellular release of COX-siRNA, inhibiting PGE2-mediated NF-κB pro-inflammatory signalling and regulating macrophage polarisation from M1 to M2. The system further promotes secretion of anti-inflammatory factors alongside VEGF and TGF-β, accelerating angiogenesis, collagen remodelling, and interfacial mineralization to achieve multi-level intervention encompassing ‘inflammation targeting-immune regulation-regeneration promotion’ (108).

#### 7.2.4 Collagen-nanohydroxyapatite scaffold antagonizing miR-138 activation

The CHA scaffold, combined with antagomiR-138/nHA and CuBG, promotes TBI healing by relieving inhibition across four key pathways to regulate macrophage polarisation: (1) The Wnt/β-catenin pathway enhances osteogenic gene expression; (2) The HIF-1α/VEGF axis promotes angiogenesis, with Cu²^+^ mildly inducing ROS to amplify signalling; (3) The ERK1/2-MAPK pathway amplifies osteogenic and β-catenin activity; (4) The BMP/SMAD pathway facilitates osteogenesis and endochondral ossification. This multi-pathway synergy, combined with an antimicrobial copper ion microenvironment, achieves rapid repair of large bone defects (109).

### 7.3 Hydrogel-related materials

#### 7.3.1 HUVECs-Exos-Fused injectable hydrogel

Owing to the inherent low metabolic activity of tendons, their minimal oxygen uptake, sparse vascularisation, reduced cellularity following rupture, and the gap created by retraction of the torn tendon, the restored function of an injured tendon rarely fully recovers its original capacity after healing (64, 110, 111). HUVECs-derived exosomes (HUVECs-Exos) incorporated into injectable hydrogels significantly enhance tendon repair. This system improves the local immune microenvironment by suppressing pro-inflammatory M1 macrophages and promoting anti-inflammatory M2 macrophage polarisation. Concurrently, it regulates the NF-κB, Wnt/β-Catenin, JAK-STAT and VEGF signalling pathways to enhance tendon stem cell proliferation, inhibit apoptosis, and support angiogenesis and tissue regeneration, thereby accelerating healing at the tendon-bone interface (112, 113).

#### 7.3.2 Mesoporous silicon dioxide hydrogel (CP@SiO_2_)

As shown in **Figure 3**, CP@SiO_2_ creates a favourable regenerative microenvironment for tendon repair by inhibiting M1 macrophage polarisation and promoting M2 macrophage polarisation. This reduces the release of pro-inflammatory factors while increasing the secretion of anti-inflammatory factors and tissue repair-related factors. Under normal conditions, GSK-3β degrades β-catenin. When GSK-3β is inhibited by ions released from the material, β-catenin escapes the degradation complex, accumulating extensively in the cytoplasm before translocating to the nucleus. Nuclearised β-catenin directly binds to NF-κB subunits, primarily p65. This interaction forms an inactive complex, preventing p65 from binding to the promoter regions of pro-inflammatory genes. Consequently, even in the presence of inflammatory stimuli within the microenvironment, the transcription factor remains ‘hijacked’ by β-catenin, preventing macrophages from initiating M1-type gene expression and thereby exerting an anti-inflammatory effect. Intranuclear β-catenin binds to TCF/LEF transcription factors, initiating transcription of downstream target genes. Growth factors and extracellular matrix components secreted by M2 macrophages further promote proliferation and differentiation of tendon stem/progenitor cells, enhancing tendon mechanical properties and functional recovery. This achieves a dual effect of inflammation suppression and tissue regeneration (114, 115).

### 7.4 Biological factors

#### 7.4.1 Exosomes

Exosomes are extracellular vesicles secreted by cells, measuring approximately 30–100 nm in diameter. Their formation commences with the invagination of the membrane of early endosomes, maturing into multivesicular bodies (MVBs) before being released extracellularly via exocytosis (116). Exosomes can regulate target cell gene expression and functional molecules through autocrine, paracrine, and endocrine modes, and significantly modulate the tendon-bone healing process by influencing macrophage (Mφ) polarisation (117). Exosomes exhibit a two-stage immunomodulatory mechanism, commencing with the local macrophage polarisation regulation phase. Exosomes suppress pro-inflammatory M1 macrophage polarisation while promoting the recruitment of anti-inflammatory M2 macrophages, thereby reducing pro-inflammatory factor levels, inhibiting cartilage matrix degradation, and diminishing scar tissue deposition. Secondly, the M2 macrophage-mediated regeneration phase occurs. M2 macrophages secrete factors such as TGF-β3 and IGF-1 to induce mesenchymal stem cells towards fibrocartilage differentiation. Concurrently, IL-10 exerts anti-inflammatory effects and promotes extracellular matrix remodelling, thereby achieving both inflammation suppression and functional tissue regeneration (118).

Exosomes are diverse in type, and those derived from different sources exhibit distinct regulatory mechanisms and modes of action within the immune microenvironment. Dendritic cell-derived exosomes (DEXs) induce M1→M2 polarisation by suppressing STAT1/NF-κB and activating the STAT6 pathway. This reduces inflammatory cytokine expression while enhancing anti-inflammatory and reparative factors, thereby promoting tendon cell differentiation, type I collagen synthesis, and inhibiting type III collagen. Consequently, DEXs enhance tendon mechanical strength (119). Tendon sheath progenitor cell exosomes (TSPC-Exos) upon phagocytosis by macrophages enrich miR-21-5p and miR-23a-3p, activating the PI3K/AKT and STAT3 pathways whilst inhibiting NF-κB. This promotes M2 polarisation, modulates the immune microenvironment and fibrosis, thereby accelerating vascularisation, ordered collagen fibre arrangement, and fibrocartilaginous transition zone regeneration (107). As illustrated in **Figure 2**, bone marrow mesenchymal stem cell exosomes (BMSC-Exo) regulate the immune microenvironment via two distinct pathways. Firstly, they suppress M1 polarisation by targeting IRF1 through miR-23a-3p, whilst inhibiting NF-κB signalling via ANXA1-FPR2 to facilitate M2 phenotypic conversion. Secondly, they modulate the Hippo/YAP signalling axis to promote VEGF-dependent angiogenesis, establishing an anti-inflammatory homeostasis. This activates the TGF-β/VEGF paracrine network, ultimately enhancing fibrocartilage regeneration and improving interfacial biomechanical properties (120, 121).

#### 7.4.2 Human-derived rosiglitazone-loaded decellularised extracellular matrix

By controlling macrophage polarisation, R-dECM facilitates tendon-bone repair. Rosiglitazone is sustainably released by R-dECM for more than seven days. In order to mitigate inflammatory reactions, rosiglitazone, a PPARγ agonist, efficiently inhibits M1 macrophage polarisation, lowers inflammatory cytokine release, and stabilizes M2 phenotype. Additionally, R-dECM preserves the M2 macrophage phenotype, supports the production of tendon-related genes (e.g., collagen I), prevents detrimental interactions between inflammatory cells and tendon stem/progenitor cells (TDSCs), and shields TDSC migration. In the end, this dual regulation system promotes tendon-bone repair and tissue regeneration by improving TDSC functional activity and optimizing the local microenvironment (122).

#### 7.4.3 Human bone marrow mesenchymal stem cell conditioned medium

Research indicates that human bone marrow-derived mesenchymal stem cell conditioned media (hBMSC-CM) significantly promote tendon-bone interface (TBI) regeneration in a rat rotator cuff injury model by modulating macrophage (Mφ) polarisation. hBMSC-CM coordinates the local immune microenvironment, inhibiting pro-inflammatory M1 macrophage polarisation while promoting anti-inflammatory/repair-oriented M2 macrophage polarisation. This effect is dependent on the Smad2/3 signalling pathway, manifested by a significant increase in phosphorylated Smad2/3 (p-Smad2/3) levels. The Smad2/3-specific inhibitor SB431542 completely blocked the macrophage-modulating effects of hBMSC-CM.

Mechanistically, amplified M2Mφ promote fibroblast recruitment to TBI through paracrine effects, accelerating the early formation of dense fibrocartilage and ultimately establishing regenerated attachment sites with ordered layered organisation. Histological and biomechanical analyses demonstrate that the newly formed tissue exhibits not only significantly improved structural remodelling but also mechanical strength approaching that of natural tendon-bone interfaces. This study reveals that hBMSC-CM initiates a cascade of tissue regeneration by modulating the immune microenvironment via the Smad2/3 signalling axis, providing a theoretical basis for immune-regulated bone-cartilage tissue regeneration strategies (123).

#### 7.4.4 EV-mediated multimodal regulation of M2 conversion

Both *in vitro* and *in vivo* studies demonstrate that the paracrine activity of mesenchymal stem cells (MSCs) and their matrix can induce macrophage (Mφ) polarisation (124–133). In mammalian models of ligament or tendon healing, MSC therapy enhances endogenous M2 macrophages and associated anti-inflammatory factors, thereby improving tissue repair (134–137). MSCs regulate wound healing by secreting multiple growth factors and cytokines, with a key mechanism influencing macrophage phenotype achieved via extracellular vesicles (EVs). Engineered EVs achieve M1/M2 rebalancing through a multi-target strategy. Membrane surface signalling: CD47 integration on EV membranes inhibits macrophage SIRPα-mediated phagocytic activation, reducing TNF-α secretion; Content delivery: EVs carrying miR-146a target IRAK1/TRAF6 to suppress the NF-κB pathway, while miR-21 promotes IL-10 release via the PDCD4/AP-1 pathway; Angiogenesis synergy: Intra-EV semaphorin 4D activates VEGFR2 phosphorylation via Plexin B1, forming positive feedback with VEGF-A secreted by M2 macrophages to promote neovascularisation. This ‘immune-vascular coupling’ strategy elevated the M2/M1 ratio at the sheep tendon-bone interface from 0.7 to 3.2, significantly accelerating tendon-bone healing (138).

### 7.5 Pharmacological factors

#### 7.5.1 Suramin

Suramin is a prescription drug used to treat human African trypanosomiasis (sleeping sickness). It possesses anti-inflammatory, anti-fibrotic and antioxidant properties, alongside other potent biological functions. The drug blocks post-injury extracellular adenosine triphosphate (ATP) activation of the immune system by inhibiting purinergic receptor signalling. It suppresses chemically induced atopic dermatitis by reducing levels of pro-inflammatory cytokines. As illustrated in **Figure 2**, suramin promotes tendinous bone healing by regulating macrophage polarisation and activating the NF-κB pathway. The drug diminishes pro-inflammatory activity by suppressing pro-inflammatory factor expression in M1 macrophages while activating their reparative functions; concurrently, it enhances the reparative capabilities of M2 macrophages. As a broad-spectrum purinergic receptor antagonist, suramin directly competitively binds to or blocks P2X7 receptors on macrophage surfaces. This step directly disrupts the pathway whereby extracellular ATP transmits the ‘inflammatory initiation signal’ intracellularly, a prerequisite for subsequent NF-κB regulation. Typically, ATP activation of P2X7 receptors induces intracellular potassium efflux and calcium influx, activating NLRP3 inflammasomes and inducing IKK phosphorylation. This leads to degradation of the NF-κB inhibitory protein IκB, releasing NF-κB (p65/p50 dimer) into the nucleus. The NF-κB dimer remains ‘locked’ in the cytoplasm, unable to enter the nucleus and bind DNA. Consequently, pro-inflammatory genes associated with M1 macrophages cannot initiate transcription. By suppressing the M1 pro-inflammatory phenotype, suramin creates an environment conducive to M2 polarisation. M2 macrophages subsequently upregulate reparative secretory functions. Furthermore, suramin promotes articular cartilage repair and angiogenesis by increasing COL1A1, COL3A1, and VEGF expression, thereby accelerating tendon-bone union. Concurrently, it synergistically enhances tendon stem cell migration, proliferation, and differentiation capacity, further driving tissue regeneration and repair (139).

#### 7.5.2 Anti-inflammatory and anti-fibrotic effects of dual-loaded budesonide and serpine1 siRNA lipid-polymer hybrid nanoparticles

Through three main methods, the dual-loaded LPN controls macrophage polarisation to facilitate tendon-bone healing: (1) Increasing the expression of the M2 marker CD206 and significantly decreasing the expression of the M1 macrophage marker CD86, which helps macrophages shift from the pro-inflammatory M1 phenotype to the anti-inflammatory and reparative M2 phenotype. (2) Dual-loaded LPN further suppresses inflammatory responses by downregulating pro-inflammatory genes and proteins. By downregulating the pro-fibrotic gene serpine1 and its associated PAI-1 protein, it concurrently slows the progression of fibrosis. (3) Dual-loaded LPNs promote tissue regeneration and repair by upregulating the expression of extracellular matrix (ECM) remodelling factors. In addition to reducing fibrosis and inflammation, this complex regulatory mechanism also fosters a favourable microenvironment for tendon-bone mending, which speeds up the healing process (140).

#### 7.5.3 Disulfiram— remodelling the inflammatory microenvironment

Disulfiram (DSF), an alcohol abuse medication approved by the FDA in 1950, has recently been found to possess potential therapeutic effects in multiple diseases beyond its action on aldehyde dehydrogenase, including anti-tumour and anti-septicaemic properties. In the latest investigation, DSF was found to significantly promote the shift of macrophages (Mφ) from the M1 phenotype to the M2 phenotype, thereby improving fibrosis at the tendon-bone interface through GSDMD-dependent pyroptosis regulation. In a mouse tendon injury model, macrophages exhibited pronounced M1 pro-inflammatory polarisation, characterised by upregulation of iNOS and TNF-α expression, activation of α-SMA+ fibroblasts, and abnormal extracellular matrix (ECM) deposition. DSF treatment markedly promoted the differentiation of CD206+ M2 macrophages while suppressing LPS/IFN-γ-induced expression of M1 markers. Animal studies revealed that oral DSF administration not only reduced F4/80^+^ macrophage infiltration but also inhibited inflammasome activation and pro-inflammatory cytokine release by blocking caspase-1/11-mediated cleavage of GSDMD-N, without affecting GSDMD precursor protein expression. Furthermore, DSF exerts cross-cell effects by modulating the biological actions of macrophage exosomes. Exosomes from DSF-pretreated BMDMs, when applied to fibroblasts, inhibited proliferation and reduced expression of α-SMA, FN, and Col-I. Concurrently, they delayed the G2/M phase of the cell cycle, thereby slowing wound healing and reducing excessive ECM deposition. This study first reveals the pathological association between macrophage pyroptosis and tendon fibrosis, proposing an innovative strategy for tendon repair intervention through a triple mechanism involving ‘immune phenotype remodelling—GSDMD inhibition—exosome regulation’ (141).

As discussed previously, the co-regulation of both macrophage phenotypes represents the ultimate objective in modulating the immune microenvironment. This encompasses the entire process of regulating inflammatory responses and numerous pathological processes, from accelerating inflammation during its initial phase to expediting healing and repair in its late stages—all of which necessitate the coordinated regulation of both phenotypes. From external stimuli such as mechanical, electrical, photothermal, and surface microtopography, to internal microenvironmental factors including reactive oxygen species (ROS), pH, and glucose levels; from fundamental hydrogels to emerging materials like engineered acoustic bodies (EABs), metallic scaffolds, and hydroxyapatite; From traditional, extensively validated nanoenzymes and hormonal therapeutics to the modern validation of millennia-old traditional Chinese medicine, evidence demonstrates that co-regulation of both macrophage phenotypes is feasible and highly effective. However, all materials and drugs must ultimately be grounded in specific studies of mechanisms and pathways. Numerous shared pathways—including NF-κB, Wnt/β-catenin, STAT3, and PI3K/AKT—not only modulate macrophage phenotypes but also regulate fibrosis, osteogenesis, angiogenesis, and antioxidant responses at the tendon-bone healing interface. Therefore, while we utilise macrophage polarisation as our entry point, our focus must extend beyond mere macrophage regulation to encompass functional studies pertinent to tendon-bone healing. This integrated approach will yield greater efficiency with less effort. Regarding existing regulatory strategies, we must pursue material innovation by advancing nanoscale and intelligent materials. Capitalising on the AI revolution, we should integrate AI with immune microenvironment-modulating materials. AI could determine drug delivery timing for materials, enabling truly personalised, round-the-clock therapeutics. Concurrently, we must leverage AI for efficient trial-and-error to identify optimal therapeutic agents, minimising adverse effects while maximising therapeutic efficacy. This approach must not only facilitate healing at the tendon-bone interface but also minimise sequelae such as fibrosis and associated complications. The rapid advancement of AI is poised to unlock entirely new perspectives in immune microenvironment regulation.

## 8 Discussion

In the inflammatory, reparative, and remodelling stages of tendon-bone repair, macrophage polarisation is especially important. M1 macrophages are crucial for triggering immunological responses, removing necrotic cells from damage sites, and promoting early inflammatory responses. On the other hand, prolonged inflammation brought on by increased M1 activation delays tendon-bone healing by preventing osteoblast development. In order to promote healing at the tendon-bone interface, macrophages must be promptly reprogrammed from pro-inflammatory M1 to anti-inflammatory M2. In addition to classifying macrophage actions according to different phases of inflammation, this paper categorises regulatory factors into three sections based on their modulation of macrophage polarisation subtypes, presented respectively in: **Table 1**: Factors Regulating M1 Macrophage Polarisation; **Table 2**: Factors Regulating M2 Macrophage Polarisation; **Table 3**: Intervening Factors Regulating M1/M2 Macrophage Polarisation Bidirectionally. At the same time, macrophage modulation frequently influences angiogenesis via several pathways, including NF-κB/Wnt/β-Catenin, which promotes tendon-bone repair in concert. According to recent studies, materials’ physical and chemical characteristics, including their surface topography, mechanical stimulation, and bioactive factor release, can successfully alter macrophage polarisation, impacting the immune microenvironment and accelerating the healing process. In order to remodel the immune microenvironment and simultaneously promote angiogenesis and osteochondral regeneration, biomaterials, bio-media, environmental factors, and drug interventions work together to influence important molecular pathways, including NF-κB, Wnt/β-catenin, STAT3, and PI3K/AKT. Nowadays, manipulating macrophage polarisation with materials has become a major area of study.

**Table 1:** Factors regulating M1 macrophage polarisation.

Serial number	Intervening factors	Molecular regulation: increase	Molecular regulation: reduction	Key pathway	References
1	Cu-MSNs	OSM, VEGF, OPG	M-CSF, RANKL	NF-κB/OSM-STAT3	(29, 142)
2	MXene nanosheets	TNF, IL-6, IL-1β	Arg-1, IL-10, CD206	NF-κB, JAK-STAT	(32)
3	R848/DTPA@DSPE-PEG NP	TNF-α, IL-6, IL-12	Tregs, M-MDSCs	TLR7/8	(33)
4	M@SINPs	ROS	CD86, TNF-α, IL-6	CD47-SIRPa	(35)
5	CCL4L2	IL-1β, TNF-α, IL-6, iNOS	/	NF-κB	(40–42)
6	CXCL12	STAT3	CD9, Pax7, BMP2, MMP2	CXCL12/CXCR4	(43)
7	CCL2	TNF, IL-6, IL-1β	Nrf2	ERK-GluN2B	(45)
8	CR-sEVs	PF4, IL-10, Arg-1	IL-1β, IL-6, TNF-α	cAMP	(48–53)
9	MHA-sEVs	Runx2, BMP2, COL I	COL III, IL-1β, TNF-α, IL-6	NF-κb	(53)
10	BIIEFS	Runx2, Col1a1, OCN	iNOS, IL-1β, CD80, TNF-α	Wnt	(51)
11	PGHE-CS4	OCN, OPN, RUNX2	CD80	PI3K/Akt, NF-κB, BMP-2	(54)
12	SF/PHBV-BBR	CD31, Ki67	p-Smad3, COX-2, iNOS	TGF-β/Smad	(55)

**Table 2:** Factors regulating M2 macrophage polarisation.

Serial number	Intervening factors	Molecular regulation: increase	Molecular regulation: reduction	Key pathway		References
1	PCLMF	Integrin β1, TGF-β3, IL-10	MMPs, iNOS, IL-1β, TNF-α		MAPK(p-JNK/p-Erk/p-p38)	(56–60, 143)
2	Wnt3a Scaffolds	MR, Arg – 1, IL – 10, β – catenin, Axin2	IL – 1, TNF-α		Wnt/β-catenin	(61)
3	MFG-E8 Scaffolds	COL1a1, tenascin - C	IFN - γ, IL - 1β, IL – 6		efferocytosis	(62, 63)
4	JTA Scaffolds	Arg-1, RANTES(CCL5)	GRO-α(CXCL1), corticosterone		VEGF-BMP-2	(64)
5	Topo-engineered Scaffolds	Scx, Collagen I, Capillary density	Collagen III, Collagen II, sGAG		/	(65)
6	BMnPs Scaffolds	BMP2, ALP, OPN, VEGF, Ang1, c-Maf, p-STAT3	TNF-α, IL-1β, IL-6, CD86		STAT3/c-Maf, VEGF/Ang1	(66, 67)
7	Mg²^+^	GAG, COL2A1, Aggrecan, AKT1	TNF-α, IL-6		Wnt/β-catenin RhoA/ROCK AKT1/PI3K	(10, 68–80)
8	TiO2 honeycombs	RhoA, Rac1, CDC42, p-MLC, p-MYPT1, BMP-2	IL-1β, TNF-α		RhoA/ROCK	(74, 83–89)
9	ASCs	IL-4 , CHI3L1, PTGR1, MMP-12	STARD13, IL-1β, iNOS, IFN-γ		VEGF	(91)
10	BCL	Runx2, Collagen I,, OCN, ALP, Arg-1	ROS, NIK, pi-p65, pi-IκB, iNOS		ROS/NF-κB	(92)

**Table 3:** Intervening factors regulating M1/M2 macrophage polarisation bidirectionally.

Serial number	Intervening factors	Molecular regulation: increase	Molecular regulation: reduction	Key pathway	References
1	ROS	p-Stat3, IL-10, IL-10, CD206, Arg1, PD-L2	p-Chk2, p-PKM2, p21, p-NF-κB, p-ERK	ROS-NF-κB/MAPK ROS-Stat3	(93–96)
2	Topology and mechanical stimulation interaction stretching	JAK1, JAK3, STAT6, CD206, Arg1, IL-10, SOX9	CD86, IL-1β, TNF-α	IL-4/JAK/STAT6 TGF-β1/Smad	(97–104)
3	Lithium-containing mesoporous silica Scaffolds	COL1A1, OCN, Runx2, VEGF, Arg-1	IL-1β, IL-6, TNF-α, iNOS	Wnt/β-catenin NF-κB MAPK	(51, 105, 106)
4	Col@PDA&T-Exos Scaffolds	VEGF, TGF-β1, CD206, Arg-1, IL-4, IL-10	iNOS, TNF-α, IL-1β	PI3K/Akt JAK/STAT	(107)
5	M2M@PLGA/COX-siRNA system	MMP2, Col I	COX-1, COX-2, Col III	COX TGF	(108)
6	AntagomiR-138-activated CHA scaffold	β-catenin, HIF-1α, SP7(Osterix), BMPR1A, BMPR1B, MMP2	miR-138	ERK1/2 Wnt/β-catenin HIF-1α BMP/SMAD	(109)
7	H-Exos-gel	CollagenI, CollagenIII;, TGF-β, IL-10, Arg1	TNF-α, IL-1β, IL-6	NF-κB/Wnt/β-Catenin	(64, 110–112)
8	CP @SiO2	SCX, Mkx, Sox2, CD44 Ki67, Col I	P53, Caspase-3/9, Col III, α-SMA, iNOS, CD86	NF-κB MAPK	(114)
9	Exosomes	IGF-1 / IGF-2 , TGF-β3, TGF-β1, IL-10	IL-1β, IL-6	TGF-β3, IGF-1	(118)
10	DEX	p-STAT6, CD206, Arg-1, TGF-β1, IL-10	TNF-α, IL-1β, iNOS, p-STAT1, CD86	STAT1/STAT6 NF-κB	(119)
11	TSPC-Exos	miR-21-5p, miR-23a-3p, miR-125b-5p, VEGF, BMP-2, Col1a1, Scx	p-NF-κB p65 TNF-α, IL-1β, IL-6	PI3K/AKT STAT3 NF-κB	(107)
12	R-dECM	COL1A1, SCX, TNMD, MKX, COL3A1, ARG1	MMP9, CCL2, TNF-α, IL-6, CD80, CSF2(GM-CSF), CSF3(G-CSF)	PPARγ/NF-κB	(122)
13	BMSC-Exos	COL2α1, CD163, Arg1, IL-10, TGF-β	IRF1, P-P65, iNOS, IL-6, TNF-α, IL-1β	miR-23a-3p/IRF1/NF-κB	(120, 121)
14	hBMSC-CM	p-Smad2/3, α-SMA, TGF-β, IGF-1, VEGF, SDF-1α	TNF-α, IL-1α, IL-1β, CD86	TGF-β/Smad2/3	(123)
15	EVs	CD163, CD206, PD-L1, PGE2, VEGF, IL-13, IL-10	iNOS, IFN-γ, TNF-α, IL-12, IL-1α, IL-1β	PGE2-EP2/EP4 NF-κB VEGF	(124–138)
16	Suramin	Ki-67, PCNA, VEGF, SCX, TNC, COL3A1, COL1A1	p-NF-κB, TNF-α, COX-2, iNOS	NF-κB	(139)
17	LPN	tPA, CD206, MMP-2, IL-4	NF-κB1, TNF-α, IL-1β, TGF-β1, Serpine1	NF-κB TGF-β1/Serpine1 MMP-2	(140)
18	DSF	GSDMD, IL-4	IL-1β, IL-1α, HMGB1, GSDMD-N	GSDMD	(141)

Biomaterials are increasingly being designed with multifunctionality in mind. Modern materials must modulate immune responses to facilitate healing in addition to providing structural support. In order to cause M2 polarisation of macrophages, functionalized biomaterials, such as nanomaterials and scaffolds containing immunomodulatory components, can locally release certain signalling molecules, including TGF-β, IL-4, and IL-10. Through interactions with macrophages, these materials enhance the immunological milieu, which aids in the resolution of inflammation and the repair of cartilage and bone. Moreover, macrophage responses are strongly influenced by the mechanical characteristics, surface topography, and rate of degradation of biomaterials. For example, through mechanosignalling, nanoscale surface features stimulate the RhoA/ROCK pathway, which in turn promotes macrophage M2 polarisation and improves cell adhesion and proliferation. Therefore, in addition to mechanical qualities and biocompatibility, material design must take into account how surface features and molecular activities can affect immune responses. Furthermore, new multipurpose materials like extracellular vesicles, drug-loaded nanoparticles, and 3D-printed scaffolds are becoming the next big thing in materials science research. By focusing on immunomodulatory factors, nano-drug delivery devices accurately control local immune responses and enhance macrophage polarisation states. Functionalized 3D-printed scaffolds optimize the repair process at the tendon-bone interface by simulating its biomechanical characteristics and promoting M1-to-M2 conversion through the phased release of growth factors or immunomodulators. The many classical and non-classical routes that control macrophage polarisation, together with common and uncommon regulatory variables, are thoroughly described in this work. It organises its study according to the timing of inflammation as the trunk, tendon-bone healing as the fruit, and macrophage polarisation controlled by different substances and factors as the branches. Particularly in-depth and comprehensive research has been done on material elements such as scaffolds, hydrogels, and exosomes. Notably, new external modulators such as extracellular vesicles formed from circadian rhythms and topological stimuli highlight the importance of physical and systemic elements in immune modulation. These findings extend our knowledge of tendon-bone healing beyond merely pharmacological treatments. Nevertheless, despite promising outcomes in small animal models, it is still difficult to apply these methods in clinical settings. First, the majority of the materials and methods now in use concentrate on altering the immunological microenvironment. Future studies should focus on precisely controlling material degradation rates, mechanical characteristics, and surface functions in order to coordinate immunological, reparative, and regenerative processes. Second, even though small animal models have shown promising results, additional validation is needed before these results can be applied to larger animal models and clinical trials. Lastly, even though previous studies have demonstrated the significance of macrophages in the healing process, more research is necessary to fully understand the intricate relationships that exist between materials and macrophages, as well as other cell types.

In conclusion, the following areas should be the focus of future research: (1) Accurate material and immune response regulation: creating intelligent biomaterials that can accurately alter macrophage polarisation at various phases; (2) Combining immune regulation and mechanical signals: Examining how immunological variables and mechanical loads work together to maximize healing at the tendon-bone junction; (3) Multidimensional material design: combining extracellular vesicles, drug delivery methods, and biomaterials to offer all-encompassing therapeutic solutions; (4) Translational research: Using clinical validation and large-animal models to assess the therapeutic applicability of current technology.

## 9 Conclusion

A dual “immune-regenerative” regulatory target for tendon-bone repair has been identified by macrophage polarisation research, and its phased intervention approach is well-positioned to get past conventional therapeutic constraints. This study summarises and discusses the interventions and potential mechanisms that affect tendon-bone healing by modulating macrophage polarisation, particularly highlighting the significant roles played by biomaterials such as scaffolds, hydrogels, and exosomes. A promising therapeutic target for tendon-bone repair is macrophage polarisation; nevertheless, successful translation necessitates collaboration between clinical orthopaedics, immunology, and biomaterials research. We may overcome this long-standing difficulty in tendon-bone integration and improve surgical results for patients with musculoskeletal injuries by developing new materials and understanding/using the “immune-regenerative axis”.
